# Dobbs-driven expansion of perinatal palliative care: a scoping review of the evidence and its limits

**DOI:** 10.1093/haschl/qxaf081

**Published:** 2025-04-15

**Authors:** Abigail B Wilpers, Kathie Kobler, Robyn Schafer, Melissa Wilpers, Molly Zeme, Janene Batten, Lucinda Canty, Scott A Lorch

**Affiliations:** Department of Family and Community Health, University of Pennsylvania School of Nursing, Philadelphia, PA 19104, United States; Research Institute, Children's Hospital of Philadelphia, Philadelphia, PA 19146, United States; Center for Fetal Care, Advocate Children's Hospital, Chicago, IL 60068, United States; Division of Advanced Nursing Practice, School of Nursing, Rutgers University, Newark, NJ 07107, United States; Department of Obstetrics, Gynecology, and Reproductive Sciences, Robert Wood Johnson Medical School, Rutgers University, New Brunswick, NJ 08901, United States; Independent researcher, Burbank, CA 91505, United States; School of Medicine, University of California, San Francisco, CA 94143, United States; Harvey Cushing/John Hay Whitney Medical Library, Yale University, New Haven, CT 06510, United States; Elaine Marieb College of Nursing, University of Massachusetts Amherst, Amherst, MA 01003, United States; Division of Neonatology, Children's Hospital of Philadelphia, Philadelphia, PA 19104, United States; Perelman School of Medicine, University of Pennsylvania, Philadelphia, PA 19104, United States

**Keywords:** reproductive health policy, palliative care, abortion

## Abstract

As abortion care restrictions increase, a growing population is continuing pregnancies complicated by life-limiting fetal conditions, making it more critical than ever to evaluate the state of the evidence in perinatal palliative care (PPC). Perinatal palliative care provides interdisciplinary, person-centered care, integrating medical management with psychosocial and bereavement support to enable values-driven decision-making. This scoping review evaluates US-based evidence on the safety, effectiveness, acceptability, and equity of PPC, assessing how these findings relate to growing abortion restrictions. Analysis of 13 studies found that US PPC programs are understudied, with limited evidence on maternal health and neonatal comfort outcomes. Studies lacked use rates for all eligible individuals, preventing assessment of overall PPC uptake. Most PPC patients reported high satisfaction, citing compassionate care, emotional support, and parental validation. However, studies lacked diversity. None examined the experience of receiving PPC due to abortion restrictions. Existing PPC evidence is limited, leaving critical gaps in safety, effectiveness, acceptability, and equity—key factors in assessing whether PPC meets its intended goals and serves diverse populations. Our review highlights that evidence is insufficient to determine whether PPC can adequately support the growing, vulnerable patient population now directed into it by policy rather than choice.

## Introduction

In the wake of the Dobbs ruling and the resulting wave of state abortion restrictions across the United States, care for individuals facing pregnancies complicated by s (LLFCs) is shifting profoundly.^1^ As prenatal detection techniques advance, more individuals and families are discovering during pregnancy that they have LLFCs, such as trisomy 18 or anencephaly, and face a significant risk of pregnancy loss, stillbirth, or infant death.^2^ Patients should be offered the opportunity to discuss care options, including terminating the pregnancy or continuing with perinatal palliative care (PPC). However, as states restrict or eliminate access to abortion, more individuals are continuing pregnancies complicated by LLFCs, making it more critical than ever to evaluate the state of the science for PPC in this evolving landscape.

Perinatal palliative care is a specialized, interdisciplinary approach that provides comprehensive care from prenatal diagnosis through labor, birth, neonatal care, and end-of-life support. It encompasses medical management of the maternal–fetal dyad alongside psychosocial, spiritual or existential, and bereavement support tailored to the unique needs of families continuing pregnancies with LLFCs.^3^ Rooted in honoring autonomy, PPC emerged to address the historically insufficient standards of care for individuals continuing pregnancies with LLFCs, ensuring they have the support needed to feel informed and empowered in their decision.^4,5^ By providing tailored, individualized care, PPC enables patients to make values-driven choices that reflect their unique priorities and goals.

Originally focused on supporting individuals who wanted minimal or no fetal/neonatal life-extending interventions, PPC has evolved into a model that integrates palliative care with curative or life-extending treatments in complex and uncertain cases, while also encompassing approaches such as early induction of labor (a method of pregnancy termination) within a palliative framework.^6,7^ Despite this evolution, policies limiting abortion care increasingly position PPC as a substitute to providing the option for abortion services.^8-14^

Unfortunately, existing reviews of PPC evidence have limited applicability to the United States because most studies were conducted in other countries where health care policies, structures, and access to resources, including abortion care, differ.^15,16^ US data often are derived from neonatal palliative care (NPC) programs, which differ from PPC programs in important ways. Specifically, NPC typically begins after birth and focuses on infants, whereas PPC begins during pregnancy and centers the care of both the pregnant person and fetus, spanning prenatal diagnosis, birth, neonatal care, and bereavement support.^16,17^ Gaps in PPC evidence underscore the need for systematic evaluation of the state of the science in US PPC to inform its continued integration into reproductive health care and policy for diverse care populations.

To address this gap, this scoping review examines existing evidence on 4 key dimensions of PPC—safety, effectiveness, acceptability, and equity—that are essential for evaluating its impact and relevance to diverse US populations.^18^ Specifically, we explore (1) safety and effectiveness, through clinical outcomes and goal-concordant care; (2) acceptability, via use rates, patient satisfaction, and qualitative insights; and (3) equity, based on the diversity and representativeness of study samples. Given the emerging nature of PPC, a scoping review is well suited to synthesize findings across study designs, map the current literature, and identify evidence gaps.^19^ Our goal is to inform policy in anticipation of the influx of patients who will be offered PPC as their only care option due to abortion restrictions.

## Data and methods

### Search strategy

The search strategy for this scoping review was designed by an expert medical librarian (J.B.) in consultation with the first author (A.B.W.) and second author (K.K.), then reviewed by a second expert searcher (Tom Mead). Databases were searched using both controlled vocabulary and synonymous free text to capture the concept of PPC. Search terms for fetal life-limiting conditions were intentionally broad due to poor indexing and variation in terminology. No limits, such as language or date range, were applied. Databases included the following: OVID Medline(R)ALL, OVID Embase, OVID PsycINFO, CINAHL, and Web of Science Core Collection. All searches were run on June 21, 2023; then updated on October 23, 2024. Supplementary efforts to identify studies included checking reference lists and contacting experts in the field. The Appendix lists all search histories. Results were uploaded to EndNote (version 21; Thompson Reuters), deduplicated, and screened in Covidence systematic review software (Veritas Health Information).

### Study selection and analysis

Eligible studies met the following inclusion criteria: (1) published in English in a peer-reviewed journal, (2) available in full text, (3) conducted in the United States, and (4) contained primary evidence of patients receiving PPC and experiences and/or outcomes of PPC. Perinatal palliative care was defined as programs including all primary components of a formal PPC consultation resulting in the development of a birth plan, access to neonatal and pediatric specialties, and perinatal support including bereavement counseling, as per the standards of the American College of Obstetricians and Gynecologists.^20^ Studies were excluded if they only assessed the perspectives of health care professionals, focused solely on bereavement, NPC, and unexpected perinatal loss.^16^

All abstracts and full texts were reviewed independently by 2 authors (A.B.W., R.S., M.W., M.Z.) to ensure rigor. Once a set of included texts was finalized by consensus, data extraction was completed to capture studies' aims, design, sample size and characteristics, outcomes assessed, and major findings (Table 1). Narrative descriptions of the findings were reviewed and confirmed by consensus. Guidelines for the Preferred Reporting Items for Systematic Reviews and Meta-Analyses Extension for Scoping Reviews (PRISMA-ScR) were followed.^21^

**Table 1.** Characteristics of the sample.

Study	Site	Aim	Design and sample size	Fetal conditions	Outcomes assessed	Major findings and conclusions	Sample demographic characteristics reported
Buskmiller et al, 2022^34^	TX	Describe outcomes of PPC at the Fetal Center of the University of Texas Health Science Center at Houston and Women's Center at Children's Memorial Hermann Hospital	Retrospective chart review (187)	Reported by category: Chromosomal Central nervous system Cardiovascular/chest Musculoskeletal Genitourinary Other	Alignment with patient care goals: counseling/plan match neonatal course/birth event and reason for mismatch and changes Neonatal: neonatal death, respiratory failure, cardiovascular instability, seizure (nonhypoglycemic), culture-proven/histologic sepsis; admissions length of stay Pregnancy: fetal demise, GA at birth, PPROM, abruption, intra-amniotic infection, postpartum hemorrhage Utilization: no. of PPC consults; no. of ultrasounds	Most families' perinatal experiences matched birth plans and expectations. Families who desired interventions used more health care resources but often did so with plans for postnatal comfort care. PPC was safe for maternal patients and equitable across racial, ethnic, and income groups.	Age Ethnicity Income Insurance Marital status Race
Côté-Arsenault and Denney-Koelsch, 2011^22^	NY	Clarify the experiences and needs of families in order to design responsive PPC services, and to establish the feasibility and acceptability of conducting intensive interviews of pregnant women and their partners during their pregnancy with an LLFC	Qualitative descriptive (8)	Trisomy 18 Hypoplastic left heart Renal agenesis Potter's sequence Multiple genitourinary malformations	Acceptability: qualitative description of parental experiences Neonatal: death shortly after birth, infant's condition better than expected Pregnancy: fetal intrapartum death, live birth Utilization: PPC referrals	Providers need to recognize patients' need to be treated as real parents, with acknowledgement of their infant as a person. Parents asked for supportive counseling to help them understand medical conditions, make informed decisions, and communicate with multiple providers. Parents want counseling that is provided with attention to continuity of care, a hopeful approach that acknowledges prognostic uncertainty, and advice that is nonjudgmental.	Age Education level Income Race
Côté-Arsenault et al, 2019^23^	Multiple, not specified	Examine the person characteristics, quality of PPC received, and parent health outcomes	Comparative mixed-methods case study (15)	Trisomy 13 Osteogenesis imperfecta Kidney, lung, and heart anomalies Hydrops Anencephaly	Acceptability: parental satisfaction and quality indicators of PPC instrument; qualitative description of patients' experiences Maternal: perception of overall health (Short-Form Health Survey, Version 2), Hospital Anxiety and Depression Scale (HADS), the Impact of Event Scale– Revised (IES-R), the Grief and Meaning Reconstruction Inventory (GMRI), “Have you received any health diagnoses since baby died?”, couple relationship status	Parents rated their general health close to good, physical health close to normal but mental health lower than the population norm. High levels of anxiety were reported in 50% of parents, whereas depression scores were normal. The experience of fetal diagnosis and infant death had a negative impact on the health of 40% of participants; however, parents could not identify what specifically caused their health problems. Most were satisfied with their PPC but some shared that original prenatal providers were not supportive of pregnancy continuation. After the infant's death, 71% reported closer/stronger couple relationships.	Age Education Ethnicity Income Race Religion & spirituality
Crawford et al, 2021^24^	Intermountain West region	Explore the experiences of women who received life-limiting fetal diagnoses during pregnancy and support from a PPC program	Qualitative descriptive, content analysis (12)	Trisomy 18 Multiple anomalies Hypoplastic left heart syndrome Hydrops fetalis Renal agenesis Turner syndrome Body stalk abnormality Skeletal dysplasia and short jaw Lobar holoprosencephaly Cloacal malformation	Acceptability: qualitative description of patients' experiences	Four themes: (1) importance of memorabilia to cope with the death and documentation of pregnancy, (2) acceptance of death as part of the pregnancy experience, (3) continued life without a child, and (4) importance of empathy throughout the process.	Age
D'Almeida et al, 2006^25^	IL	Demonstrate the feasibility of establishing a perinatal hospice program in a community perinatal referral center	Retrospective chart review (28)	Trisomy 18 Hydrops Acrania Anencephaly Renal agenesis Body stalk anomaly Triploidy Achondroplasia type III Thanatophoric dwarf Complex CHD	Maternal: postpartum readmission Neonatal: days of life Pregnancy: fetal demise, live births, preterm births, mode of birth, infection, operative complications, need for blood products Utilization: % uptake after offer of PPC	Twenty-eight newborns prenatally diagnosed with lethal anomalies were eligible for a pilot hospice program at Rockford Memorial Hospital. Comprehensive care in a comforting community setting was provided to the 75% of families who chose this option. No maternal morbidity resulted. In the 75% of infants who were born alive, survival ranged from 20 minutes to 256 days.	None
Farmer et al, 2023^26^	OH	Evaluate outcomes and PPC continuity in infants born to families who received PPC at a quaternary care pediatric hospital, and to identify targets to improve care continuity	Retrospective chart review (181)	Reported by category: Genetic Multiple congenital anomalies Renal/GU Neurological Chest Cardiac HEENT Other	Neonatal: infant death, admission length Pregnancy: fetal demise, GA at birth; location of birth Utilization: postnatal palliative care involvement (rate and time to consult); location of care	Continuation of palliative care after birth in families who received perinatal palliative care is inconsistently achieved. Creating reliable systems for PPC continuity will depend on location of care.	None
Kamrath et al, 2019^27^	MN	Describe infant interaction with the health care system and by gaining deeper understanding of the maternal experience after being offered PPC	Mixed methods (27)	Chromosomal abnormalities Skeletal dysplasia Central nervous system malformation Severe cardiac anomalies Severe renal anomalies Hydrops fetalis Complex congenital diaphragmatic hernia Metabolic Severe airway anomalies	Acceptability: qualitative description of patients' experiences Maternal: Complicated Grief Questionnaire Neonatal: ICU days; days from DNAR to death; death in NICU; death following discontinuation of CPR Utilization: % of patients offered PPC and uptake; aggressive life-sustaining treatment provided; withdrawal from life-sustaining treatment; no. of invasive procedures	Between 2011 and 2014, 70% of eligible patients were offered PPC; of those, 66% enrolled. No participants met criteria for complicated grief. Differences in infant's end-of-life trajectory, including location of death, number of invasive procedures, and death in the setting of withholding vs withdrawing life-sustaining treatment. Without a PPC plan, the default treatment for infants with LLFC conditions is likely to be invasive and painful with often minimal likelihood of long-term survival. Analysis of interview and focus group data revealed 3 themes: care, choice, and legacy.	Age Ethnicity Medical assistance Race Relationship status
Lathrop and VandeVusse, 2011^28^	US-based; not otherwise specified	Explore the experiences of perinatal hospice mothers, to gather knowledge useful to health professionals, and to guide future research	Qualitative narrative analysis (15)	Anencephaly Mosaic trisomy Trisomy 18 Trisomy 13 Osteogenesis imperfecta TII Renal agenesis Holoprosencephaly Urorectal septal malformation sequence	Acceptability: qualitative description of patients' experiences Neonatal: duration of life after birth	Participants identified as mothers and identified their fetuses or newborns as babies. Mothers valued caring for and interacting with their babies. Health professionals who affirmed their status as mothers, the value of their babies, and the significance of their losses were perceived as supportive. Invalidating attitudes and behavior caused significant distress among mothers.	Age Education Ethnicity Income Race
Marc-Aurele and Nelesen, 2013^29^	CA	To learn: (1) who is referred for PPC at San Diego Hospice; (2) what happens after referral to PPC; and (3) what happens after birth for this population	Retrospective chart review (66)	Trisomy 13 Trisomy 18 Anencephaly Other chromosomal Other neurological Other causes of pulmonary hypoplasia Unrepaired congenital heart disease Unknown	Pregnancy: mode of birth; live birth Neonatal: duration of survival after birth Utilization: GA at referral; extent of prenatal care; time from referral to start of palliative services; duration of palliative services for mother; how many PPC visits made prior to birth; how many women choose aggressive interventions vs comfort care at birth; whether birth plan created; whether final arrangements or ceremonies at birth planned; resuscitation at birth; no. of NICU admissions; hospice admissions	PPC was given over a mean of 45 (0-132) days and 3 (0-12) visits prior to birth. Most women completed a birth plan prior to birth and chose PPC only. Forty-one deliveries resulted in a liveborn infant. Twelve liveborn infants survived past 72 hours and were admitted to pediatric hospice care. There was a 2-month gap in time between potential diagnosis and referral for PPC. One-third of women met with the palliative care team only once or twice prior to birth, indicating a need for earlier referral to provide more comprehensive care.	Age Ethnicity Father of the baby involved Insurance payor Marital status Others in the home Prenatal care Previous children Spirituality
Marc-Aurele et al, 2018^30^	CA	To determine (1) how many high-risk pregnancies referred to UCSD Medical Center have potentially life-limiting fetal diagnoses; (2) the outcome for pregnancies with potentially life-limiting fetal diagnoses: number of therapeutic abortions, intrauterine fetal demises, neonatal live born, and survival for live births; (3) how many high-risk pregnancies at UCSD with potentially life-limiting fetal diagnoses were referred to PPC	Retrospective chart review (332)	Trisomy 13/18 Other genetic defects Anencephaly Other neurologic defects Pulmonary hypoplasia Hydrops Congenital heart defects Multiple anomalies Other	Pregnancy: fetal demise, therapeutic abortion, live birth Neonatal: duration of infant survival Utilization: referrals from fetal care center to PPC; type of care received	The pregnancy outcome was determined in 95% cases: 56% therapeutic abortion, 16% intrauterine fetal demise, and 23% live birth. Only 11% of cases were referred to PPC. The vast majority of women with potentially life-limiting fetal diagnoses are not referred to PPC. Evaluation of how to integrate palliative care into high-risk obstetrics is needed.	Age Ethnicity Marital status Previous pregnancies Previous children Race
Parravicini and Lorenz, 2014^31^	NY	Describe the neonatal outcomes of a case series of infants who were prenatally diagnosed with potential LLFCs and to whom individualized comfort measures were offered	Retrospective chart review (49)	Renal agenesis Severe renal hypoplasia Hydrops Trisomy 21 Polycystic kidneys PUV Hypoplastic kidney Conjoined twins w/inoperable CHD Limb–body wall complex	Neonatal: diagnostic accuracy; age at death Pregnancy: GA at birth Utilization: type of care chosen (intensive vs comfort once born, types of comfort care services)	Diagnostic accuracy was the principal determinant of outcomes. Provision of intensive care did not prevent death of infants affected by life-limiting conditions nor prolong life compared with that of infants treated with individualized comfort measures. Further prospective studies are needed to objectively evaluate the state of the infants' comfort and the grieving experience of the family to advise best practice.	None
Tucker et al, 2021^32^	MO	To document the broad provision of PPC at 1 institution, with a focus on describing the patients served, patient outcomes, and the attitudes of neonatologists	Retrospective chart review (430)	Hypoplastic left heart syndrome Congenital diaphragmatic hernia Encephalocele Trisomy 18 Trisomy 13 Bilateral renal dysplasia Congenital pulmonary airway malformation Severe intrauterine growth restriction Sacrococcygeal teratoma	Pregnancy: fetal demise; location of birth Neonatal: survival time and number of infants surviving to discharge and home hospice Utilization: use of PPC services over time; no. of perinatal consults; use of comfort care vs resuscitation efforts (changes after birth); duration of program follow-up	Services increased from 1 PPC consult in 2010 to 102 in 2018. A total of 430 mothers received PPC consultation. Of the 390 liveborn infants, 172 died, 48 received comfort care only, and 19 survived to discharge home with hospice.	None
Winn et al, 2018^33^	WI	The study aimed to examine all trisomy 13/18 diagnoses at the center by (1) describing parental goals after diagnosis; (2) outlining the prenatal counseling provided; (3) documenting parental decisions post-counseling; (4) exploring trends in referrals, decisions, and counseling over time; and (5) analyzing perinatal outcomes for families who chose alternatives to termination	Retrospective chart review (152)	Trisomy 13 Trisomy 18	Acceptability: qualitative descriptions of counseling from medical records, alignment with goals (patient goals at initial visit; options offered) Pregnancy: mode of birth; fetal demise Neonatal: days of life; location of care Utilization: specialists seen; PPC uptake (including induction with palliative care vs expectant management) compared with termination; infant interventions	Out of 152 pregnancies, 55% were terminated. Twenty percent chose induction PPC, 20% chose expectant management, 2% chose full interventions, and 3% were lost to follow-up. Counseling was based on original parental goals. Fifty percent of expectant management cases resulted in a live birth. Women who chose neonatal interventions had a live birth in 100% of the cases. There were no long-term survivors. The majority of patients who continue their pregnancy after an LLFC diagnosis desire expectant management with palliative care. A live birth can be expected at least half of the time.	Age Parity Other living children present Additional complicating conditions Marital status Religion

Sample sizes do not necessarily reflect the number of patients who underwent PPC, as some opted for different care paths.

Abbreviations: CHD, coronary heart disease; CPR, cardiopulmonary resuscitation; DNAR, Do Not Attempt Resuscitation; GA, gestational age; GU, genitourinary; HEENT, head, eyes, ears, nose, and throat; ICU, intensive care unit (utilization); LLFC, life-limiting fetal condition; NICU, neonatal intensive care unit (admissions); PPC, perinatal palliative care; PPROM, preterm premature rupture of membranes; PUV, posterior urethral valve; UCSD, University of California, San Diego.

## Results

**Figure 1.**
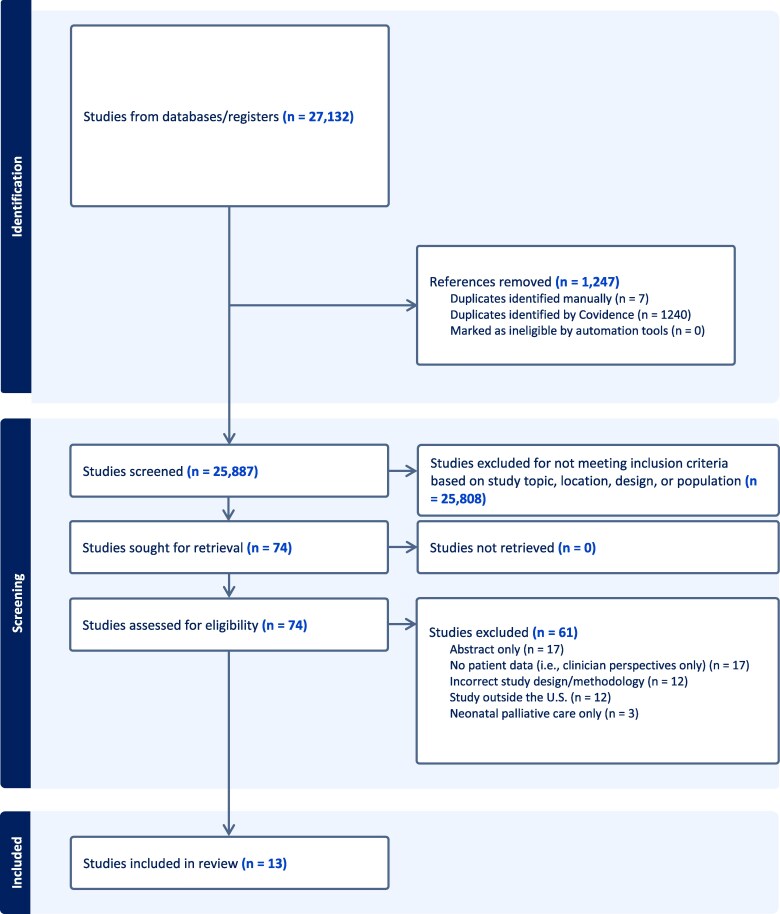
PRISMA-ScR flow diagram of study selection. Guidelines for Preferred Reporting Items for Systematic Reviews and Meta-Analyses Extension for Scoping Reviews (PRISMA-ScR) were followed.

We identified 25 887 records (Figure 1). After removing duplicates and screening for relevance, 74 full-text articles were assessed for eligibility. Of these, 13 studies were included in our final analysis (Table 1). These studies were published from 2006 to 2023 and conducted prior to the Dobbs ruling. Studies were conducted in a diverse range of locations across the United States, including the Northeast (NY), Midwest (IL, OH, MN, MO, WI), and Western (CA, Intermountain West) states. Authorship composition varied across studies, with the majority including representation from at least 2 clinical areas—typically palliative care, neonatology, obstetrics, or nursing. Notably, social workers, chaplains, psychologists, and ethicists were rarely represented in authorship, despite their central roles in PPC delivery.

Information about the palliative care programs was often missing or limited to the minimal details of inclusion criteria. Significant variation was noted in patient eligibility, service scope, timing and duration of support, and inclusion of long-term follow-up or community-based services, with most focusing on hospital-based care.

The study design and methodology included 8 retrospective chart reviews, 3 qualitative studies, and 2 mixed-methods studies. Studies aimed to identify the use of PPC services, explore patients' experiences and satisfaction, and evaluate pregnancy and maternal–child health outcomes. Sample sizes ranged from 8 to 430, with 69% of studies including fewer than 50 participants. The LLFCs reported spanned multiple types of congenital anomalies, with trisomy 18 being the most common.

### PPC safety and effectiveness

Unlike other forms of palliative care, PPC occurs in the setting of a maternal–fetal dyad, where a medically healthy pregnant individual is navigating the risks of continuing a pregnancy with an LLFC. In addition, PPC services may include specialized teams providing routine obstetric, neonatal, and behavioral health care. As such, it is important to assess safety outcomes associated with PPC.

Neonatal outcomes were the most frequently measured (85%), followed by pregnancy outcomes (69%) and maternal health outcomes (30%) (Table 1). The evidence used to assess PPC effectiveness included factors related to how well PPC addressed medical, emotional, and practical needs of the pregnant patient. Findings are presented here in order of available evidence from the most to least comprehensive available data.

#### Neonatal outcomes

Neonatal outcomes were measured by 11 studies and included admission length of stay, location of care and death (neonatal intensive care unit [NICU] vs home), death following life-sustaining interventions, and prenatal diagnostic and prognostic accuracy.^22,25-34^ Most studies explored solely the duration of infant life.^25,27-30,33^

In their retrospective review of 49 infants prenatally diagnosed with LLFCs in New York, Parravicini et al^31^ noted that diagnostic accuracy was the principal determinant of outcomes, and that provision of intensive care did not prevent death or prolong life compared with infants treated with comfort measures. Buskmiller et al^34^ conducted the only study to specifically examine neonatal symptoms, focusing on a Texas cohort of 187 families receiving PPC. The study identified respiratory failure, cardiovascular instability, and seizures as key symptoms, noting higher prevalence in infants who received life-sustaining interventions compared with those provided comfort care.^34^ No studies used a validated measure of neonatal comfort.

Qualitative studies examined neonatal outcomes based on parents' perceptions, highlighting the centrality of parents' desires for their infants to be comfortable.^22-24,27-29,33^ In 1 study, participants reported that they appreciated when their infants appeared “peaceful” and “free from suffering.”^27^ However, some parents felt that neonatal care did not align with their goals for their infant's quality of life.^29^

#### Pregnancy outcomes

Nine studies reported pregnancy outcomes, such as gestational age at birth, mode of birth, and whether the pregnancy resulted in abortion, fetal demise, or live birth.^22,25,26,29-34^ Only 2 studies specifically examined pregnancy complications, with different results.^25,34^ While no differences in pregnancy complications were noted in a study of 28 patients,^25^ a larger study found higher rates of infection and hemorrhage in the PPC patients who chose any monitoring or postdelivery intervention (9% vs 22%; *P* = .02); however, this was confounded by mode of birth.^34^ Overall, 14% of PPC participants experienced intra-amniotic infection or hemorrhage.^34^

Two studies examined how pregnancy outcomes aligned with patients' goals.^33,34^ Winn et al^33^ found that the goal of meeting the infant alive was achieved in 50% of vaginal births and 87% of cesarean deliveries. Similarly, Buskmiller et al^34^ reported that 89% of births aligned with families' plans and clinicians' expectations, although families choosing interventions had more mismatches (19% vs 2%) and were more likely to change plans (24% vs 6%) than those who chose comfort care.

#### Maternal health outcomes

Four studies quantitatively measured maternal health outcomes, including antepartum and postpartum physical and mental well-being.^23,25,27,34^ Côté-Arsenault et al^23^ conducted a mixed-methods study in North Carolina with 15 African-American and Latino parents who underwent PPC, and found that overall health perceptions were rated “good” and physical health was close to the population norm.

Two studies measured maternal mental health outcomes.^23,27^ Kamrath et al^27^ assessed maladaptive symptoms of loss and found that no participants met the criteria for complicated grief. Timing of these assessments was not specified. Côté-Arsenault et al^23^ assessed maternal mental health outcomes 6 to 36 months after perinatal death. Nearly half of the participants reported elevated anxiety, while depression scores remained within the normal range. No significant post-traumatic stress disorder symptoms were found; however, several participants indicated the emergence of new, unspecified mental health diagnoses since their infant's death.^23^

Three qualitative and 1 mixed-methods study provided descriptive insights into maternal health outcomes, focusing primarily on mental health.^22-24,27,28^ Patients reported that PPC services, like being supported in bathing and dressing a deceased infant, helped with coping and affirmation of maternal identity.^22^ Early PPC involvement helped patients feel more prepared and less overwhelmed. Participants shared that PPC was “crucial for my mental health”^29^ and made them feel “understood and supported.”^24^

### PPC acceptability

Evidence on PPC acceptability included the following: (1) overall use including referral and use rates, timing of referrals and initiation of care, and barriers to accessing preferred care; (2) use of specific services within PPC programs; and (3) assessments of patient satisfaction. Collectively, these dimensions underscore how structural and interpersonal elements shape patients' ability and willingness to engage with and benefit from PPC services.

#### Use among eligible and referred populations

Of the 10 studies reporting PPC use, 2 included both the total eligible patient volume alongside the proportion referred for and/or receiving PPC services, which are both necessary metrics to assess use rates.^25,27,30,31,33^ The study by Marc-Aurele et al^30^ was unique in comprehensively assessing all eligible individuals, those referred, and those who utilized PPC. They found that only 11% were referred for PPC, and of the 36 women referred, 26 received care. Winn et al^33^ found that, of the 152 individuals with pregnancies complicated by fetal trisomy 13 and 18, 20% opted for early induction of labor (ie, pregnancy termination) with palliative care, and 20% chose expectant management (ie, pregnancy continuation) with palliative care. The remaining 6 studies focused solely on PPC use within populations already referred for or offered PPC services at single sites. Use rates ranged from 62% to 75%, with sample sizes from 27 to 49.^25,27,31^ Among the 5 studies reporting PPC program volumes, patient volumes ranged from 9 to 69 patients per year.^15,24,26,29,32^

Three studies examined length of time from diagnosis of an LLFC to referral or onset of PPC care.^15,22,29^ Two studies reported a 2-month gap from diagnosis to referral for PPC.^26,29^ In their qualitative study, Côté-Arsenault and Denney-Koelsch^22^ reported that all the parents interviewed were “eventually” referred to PCC services by their third trimester, but many wished it had occurred closer to diagnosis. Other qualitative studies highlighted reasons for referral delays, with many individuals describing needing to educate both themselves and their providers about PPC, and fight for access to these services, often requiring changing providers or health care systems.^22,23,27^

In many cases, individuals were offered and declined termination of pregnancy many times.^28-30^ Few studies assessed patients' initial preferences for their care compared with the care they were offered and received.^15,33^ Barriers to accessing abortion services, such as insurance delays or refusals, influenced enrollment in PPC, with qualitative findings indicating that some individuals were directed toward PPC after being discouraged from seeking abortion care.^30,33^ However, limited details were provided related to these cases.

#### Use of specific services

Differences in how services were defined, measured, and used further limited the ability to make cross-study comparisons of acceptability.

Services included prenatal/postnatal consultations, ultrasounds, antepartum and birth admissions, presence or absence of fetal heart rate monitoring during labor, birth methods, NICU admissions, neonatal treatments, comfort measures, and home visits.^15,25-27,29,31-33^ Most studies did not detail factors influencing use, such as institutional practices vs patient preferences, and did not clarify whether use reflected patients opting into or out of standard perinatal services. Qualitative data indicated that some PPC patients may request extra ultrasounds for lasting memories,^28,30,33^ while others decline standard ultrasounds to avoid emotional distress.^28,33^ Similarly, in most studies, it was unclear how options for intended mode of birth were presented and chosen.

#### Patient satisfaction

Approximately half of the studies assessed patient satisfaction.^22-24,27-29,33^ One study measured satisfaction using the only validated instrument in PPC, and found strong satisfaction during prenatal, intrapartum, and postnatal time periods.^23^ Qualitative insights revealed that many parents found PPC's compassionate and supportive approach to be a profound relief as evidenced by statements like, “They made me feel like I and my baby mattered.”^23^

Most studies assessing patient satisfaction relied on qualitative data, which highlighted the value of memorabilia, empathy and support from health care professionals, and validation of parental roles.^22,24,27,28,33^ One participant expressed gratitude for the support she was given to spend as much uninterrupted time with her infant as possible, saying, “I slept with [my baby]. Just held him real close to me … to savor every moment I could.”^28^ Perinatal palliative care also served as a crucial connection point to other LLFC families, allowing them to share support, validation, and community.^24,28^

Although participants generally described their PPC experiences positively, some studies reported dissatisfaction. One study revealed that some intrapartum and postpartum clinicians questioned the appropriateness of birth plans made with PPC providers, particularly regarding post-death interactions. One PPC patient described staff avoiding eye contact, which she felt stemmed from anticipation of her infant's death.^22^ Additionally, some parents faced difficulties when their infants survived longer than expected and were unprepared to provide infant care at home.^28^

### Equity in PPC evidence

While we could not directly measure equity in access or delivery of PPC across all studies, sample characteristics—especially in the many retrospective chart reviews—offered important insight into which populations have received PPC services. This allowed us to assess whether the existing evidence base reflects a diverse patient population or is limited in ways that affect its external validity and relevance for equitable care. We evaluated this by examining how sample characteristics were reported, the diversity of study cohorts, and the impact of these factors on patients' experiences with PPC.

#### Reporting of sample characteristics

Sociodemographic characteristics of the study samples were rarely reported (Table 1). Four studies (31%) did not include any demographic details.^25,26,31,32^ Of the 9 studies that did, age was the most commonly reported variable (88%),^15,22-24,27-29,33^ followed by race and/or ethnicity (67%),^15,22,23,27-29^ income level (44%),^15,22,23,28^ relationship status (44%),^15,27,29,33^ educational level (33%),^22,23,28^ insurance (33%),^15,27,29^ and religion/spirituality (33%).^23,29,33^ Less than 10% of studies reported any other sociodemographic variables.

#### Diversity and representation

Participant ages ranged from 14 to 46 years, with a median maternal age of 30 years across studies.^15,22-24,27-29,33^ Of the 6 studies that reported race and ethnicity, participants were primarily White non-Hispanic, making up an average of 54% of each sample from a range of 13% to 88%, followed by Hispanic or Latina (50%, 0%–65%), Black or African American (13%, 0%–40%), and Asian (5%, 0%–13%). No other racial or ethnic groups were listed as participants. Variation in approaches to assessing both age and race/ethnicity made it difficult to compare across studies.

One quarter of studies excluded participants who did not speak English.^23,24,28^ Annual income levels, reported in 4 studies, ranged widely from $0 to $200 000.^15,22,23,28^ Most participants were married or partnered (62%–93%),^15,27,29,33^ and completed high school or college education.^22,23,28^ Three studies captured insurance type, revealing a higher prevalence of public insurance among PPC patients.^15,27,29^ Most participants identified as Christian (57%–82%; mean: 73%),^23,29,33^ and in 1 study all participants were “somewhat” to “very religious.”^23^

#### Influence of sociodemographic factors

Only 1 study assessed the influence of sociodemographic factors on PPC outcomes, which found no significant differences in care outcomes, including alignment between birth plans and events, across racial, ethnic, and income groups.^34^ Qualitative findings provided contextual insights into sociodemographic influences on PPC access, experiences, and outcomes. One study included accounts of needing to relocate to receive PPC,^23^ while another described experiences of insurance delays and refusals.^33^ One study specifically examined the experiences and outcomes of African-American and Latino bereaved parents but did not explore whether these experiences were shaped by racialization or differed from those of other racial groups.^23^

## Discussion

While extensive literature describes the development of PPC, its components, and care delivery, this scoping review is the first to synthesize evidence on the safety, effectiveness, acceptability, and equity of PPC within the United States. This review found that the safety and effectiveness of US PPC programs remain underexplored, with most research lacking comprehensive measures of maternal health and neonatal comfort. Evidence on PPC acceptability is similarly limited, as most studies did not report referral or use rates among all eligible individuals, making it impossible to accurately assess uptake. While some studies noted access barriers such as insurance limitations, these issues were not explored in depth. Patient satisfaction scores and qualitative insights indicated that most patients who received PPC found it valuable, with high satisfaction driven by compassionate care, emotional support, and validation of parental roles. However, equity in PPC remains a significant concern, as most study participants were White, Christian, and married. This lack of diversity raises concerns about the inclusivity of PPC and limits the generalizability of existing evidence.

One of the most significant gaps in PPC research is the lack of comprehensive data on neonatal and maternal health outcomes, limiting the ability to assess effectiveness and safety. The most consistently reported outcomes of PPC programs in the United States are gestational age at birth and neonatal survival duration—measures that are not typically influenced by PPC. These studies noted important gaps in care delivery, such as the need to better prepare families for longer-than-anticipated neonatal survival. More meaningful neonatal outcomes to measure would include assessments of neonatal comfort and symptom management in these PPC programs.^35^ The narrow focus on neonatal outcomes also overlooks critical maternal health risks in pregnancies with LLFCs, such as hemorrhage,^34,36^ prolonged labor,^34,36^ and mirror syndrome.^37^ Among the limited studies examining maternal outcomes, the largest in this review reported a 14% postpartum hemorrhage rate, far exceeding the general population rate (3%–8%).^38^ By comparison, hemorrhage after second-trimester dilation and evacuation (a method of pregnancy termination) occurs in 0.09%–1% of cases.^39^ This highlights the critical need for broader investigation into maternal risks related to continuing pregnancies with LLFCs in US PPC programs.

The variability in PPC program development across US medical centers likely contributes to the limited evidence base.^2^ As with pediatric palliative care a decade ago, PPC programs face significant challenges related to interdisciplinary team formation, provider education, and institutional support.^2^ These constraints impact both the availability of PPC services and the ability to generate evidence—underscoring the need for structural investment to advance the field.

In a post-Dobbs landscape, perhaps the most important finding of this review is the absence of studies examining where PPC may have been received but was not preferred.^40^ Of the approximately 6 million pregnancies in the United States each year—including nearly 1 million abortions prior to Dobbs— approximately 70% of those involving LLFCs were previously terminated.^1,41^ As a result, current restrictions are likely to result in thousands more patients continuing LLFC pregnancies due to legal constraints rather than personal choice. Palliative care organizations, including the National Coalition for Hospice and Palliative Care, uniformly oppose the Dobbs ruling, emphasizing that it undermines patient autonomy, interferes with patient–clinician relationships, and restricts access to evidence-based, person-centered care, particularly for marginalized populations.^42^ Leading experts in PPC highlight how laws like Dobbs force the continuation of pregnancy under the guise of providing PPC while simultaneously undermining its principles.^43^ “Born Alive” bills contradict PPC's focus on parental choice, comfort care, and quality of life.^44^ These policies not only limit families' autonomy but also conflict with the ethical foundation of PPC, centered on individualized, compassionate decision-making.

This review has several limitations. The lack of comprehensive data on eligibility, referrals, and use, as well as maternal outcomes and sociodemographic characteristics, constrained our ability to assess acceptability, safety, and equity in detail. In addition, reliance on a small number of studies, many with qualitative or retrospective designs, highlights the emerging nature of the evidence base and need for additional research.

## Conclusion

While PPC is widely recognized as a valuable care option, existing research is limited in the United States, with significant gaps in evidence of safety, effectiveness, acceptability, and equity. This research is essential for evaluating how well PPC achieves its intended goals and meets diverse patients' needs. Notably, no studies have examined experiences of individuals who received PPC when they may have preferred abortion. This gap is increasingly relevant as more people continue pregnancies with LLFCs due to abortion restrictions. We urge policymakers to consider how abortion restrictions fail to align with evidence-based practices and conflict with PPC's core principles.

## Supplementary Material

qxaf081_Supplementary_Data

## References

[1] Lorch SA, Wilpers A, Montoya-Williams D. The Dobbs decision and pediatric healthcare: preparing for unintended consequences. Pediatr Res. 2024;97(1):3–5. 39578625 10.1038/s41390-024-03725-z

[2] Denney-Koelsch E, Black BP, Côté-Arsenault D, Wool C, Kim S, Kavanaugh K. A survey of perinatal palliative care programs in the United States: structure, processes, and outcomes. J Palliat Med. 2016;19(10):1080–1086. 10.1089/jpm.2015.0536 27559768

[3] Denney-Koelsch EM, Côté-Arsenault D. Perinatal Palliative Care. Cham, Switzerland: Springer; 2020.

[4] Carter BS . An ethical rationale for perinatal palliative care. Semin Perinatol. 2022;46(3):151526. 10.1016/j.semperi.2021.151526 34839940

[5] Chitty LS, Barnes CA, Berry C. For debate: continuing with pregnancy after a diagnosis of lethal abnormality: experience of five couples and recommendations for management. BMJ. 1996;313(7055):478–480. 10.1136/bmj.313.7055.478 8776321 PMC2351873

[6] Wilkinson D, Bertaud S, Mancini A, Murdoch E. Recognising uncertainty: an integrated framework for palliative care in perinatal medicine. Arch Dis Child Fetal Neonatal Ed. 2024;110(3):236–244. 10.1136/archdischild-2024-327662 39567213

[7] Leuthner S, Jones EL. Fetal concerns program: a model for perinatal palliative care. MCN Am J Matern Child Nurs. 2007;32(5):272–278. 10.1097/01.NMC.0000287996.90307.c6 17728587

[8] Utah Criminal Code . Abortion prohibition, 76-7a-201. Accessed February 1, 2025. https://le.utah.gov/xcode/Title76/Chapter7a/76-7a-S201.html

[9] Kentucky Legislature . Kentucky House Bill 467. 2024. Accessed February 1, 2025. https://www.aclu-ky.org/en/legislation/hb-467-forced-nonviable-pregnancy-bill

[10] North Carolina General Assembly . North Carolina Senate Bill 20. 2023. Accessed February 1, 2025. https://www.ncleg.gov/Sessions/2023/Bills/Senate/PDF/S20v5.pdf

[11] Texas Legislature . Texas Senate Bill 1033. 2019. Accessed February 1, 2025. https://legiscan.com/TX/text/SB1033/id/2021204

[12] Oklahoma Legislature . Oklahoma House Bill 2684. 2014. Accessed February 1, 2025. http://www.oklegislature.gov/BillInfo.aspx?Bill=hb2684&Session=1400

[13] Nebraska Legislature . Nebraska Legislative Bill 506. 2017. Accessed February 1, 2025. https://nebraskalegislature.gov/bills/view_bill.php?DocumentID=30700

[14] Indiana General Assembly . Indiana House Enrolled Act No. 1337. 2016. Accessed February 1, 2025. https://iga.in.gov/legislative/2016/bills/house/1337

[15] Buskmiller C, Calhoun BC. A scoping review of perinatal palliative care: allowing parents to be parents. Am J Perinatol. 2023;40(12):1373–1377. 10.1055/s-0041-1740251 34856607

[16] Dombrecht L, Chambaere K, Beernaert K, et al Components of perinatal palliative care: an integrative review. Children. 2023;10(3):482. 10.3390/children10030482 36980040 PMC10047326

[17] Balaguer A, Martín-Ancel A, Ortigoza-Escobar D, Escribano J, Argemi J. The model of palliative care in the perinatal setting: a review of the literature. BMC Pediatr. 2012;12(1):1–8. 10.1186/1471-2431-12-25 22409881 PMC3320524

[18] Lobb R, Colditz GA. Implementation science and its application to population health. Annu Rev Public Health. 2013;34(1):235–251. 10.1146/annurev-publhealth-031912-114444 23297655 PMC3901430

[19] Munn Z, Peters MDJ, Stern C, Tufanaru C, McArthur A, Aromataris E. Systematic review or scoping review? Guidance for authors when choosing between a systematic or scoping review approach. BMC Med Res Methodol. 2018;18(1):143. 10.1186/s12874-018-0611-x 30453902 PMC6245623

[20] American College of Obstetricians and Gynecologists' Committee on Ethics . Perinatal palliative care. Obstet Gynecol. 2019;134(3):e84–e89. 10.1097/AOG.0000000000003425 31441826

[21] Tricco AC, Lillie E, Zarin W, et al PRISMA Extension for Scoping Reviews (PRISMA-ScR): checklist and explanation. Ann Intern Med. 2018;169(7):467–473. 10.7326/M18-0850 30178033

[22] Côté-Arsenault D, Denney-Koelsch E. “My baby is a person”: parents' experiences with life-threatening fetal diagnosis. J Palliat Med. 2011;14(12):1302–1308. 10.1089/jpm.2011.0165 22077542

[23] Côté-Arsenault D, Denney-Koelsch EM, McCoy TP, Kavanaugh K. African American and Latino bereaved parent health outcomes after receiving perinatal palliative care: a comparative mixed methods case study. Appl Nurs Res. 2019;50:151200. 10.1016/j.apnr.2019.151200 31735485

[24] Crawford A, Hopkin A, Rindler M, Johnson E, Clark L, Rothwell E. Women's experiences with palliative care during pregnancy. J Obstet Gynecol Neonatal Nurs. 2021;50(4):402–411. 10.1016/j.jogn.2021.02.009 PMC8286290 33775641

[25] D’Almeida M, Hume RF, Lathrop A, Njoku A, Calhoun BC. Perinatal hospice: family-centered care of the fetus with a lethal condition. J Am Phys Surg. 2006;11(2):52–55.

[26] Farmer ZJ, Palmaccio-Lawton SJ, Flint HA, Whitford B, Thienprayoon R, Nee K. Fetal outcomes and continuity in perinatal palliative care patients at a quaternary care pediatric hospital. J Perinatol. 2023;43(7):889–894. 10.1038/s41372-023-01664-x 37005452

[27] Kamrath HJ, Osterholm E, Stover-Haney R, George T, O’Connor-Von S, Needle J. Lasting legacy: maternal perspectives of perinatal palliative care. J Palliat Med. 2019;22(3):310–315. 10.1089/jpm.2018.0303 30388063

[28] Lathrop A, VandeVusse L. Affirming motherhood: validation and invalidation in women's perinatal hospice narratives. Birth. 2011;38(3):256–265. 10.1111/j.1523-536X.2011.00478.x 21884234

[29] Marc-Aurele KL, Nelesen R. A five-year review of referrals for perinatal palliative care. J Palliat Med. 2013;16(10):1232–1236. 10.1089/jpm.2013.0098 24003992

[30] Marc-Aurele KL, Hull AD, Jones MC, Pretorius DH. A fetal diagnostic center's referral rate for perinatal palliative care. Ann Palliat Med. 2018;7(2):1785–1791. 10.21037/apm.2017.03.12 28595435

[31] Parravicini E, Lorenz JM. Neonatal outcomes of fetuses diagnosed with life-limiting conditions when individualized comfort measures are proposed. J Perinatol. 2014;34(6):483–487. 10.1038/jp.2014.40 24651733

[32] Tucker MH, Ellis K, Linebarger J. Outcomes following perinatal palliative care consultation: a retrospective review. J Perinatol. 2021;41(9):2196–2200. 10.1038/s41372-021-00966-2 33597742

[33] Winn P, Acharya K, Peterson E, Leuthner S. Prenatal counseling and parental decision-making following a fetal diagnosis of trisomy 13 or 18. J Perinatol. 2018;38(7):788–796. 10.1038/s41372-018-0107-x 29740195

[34] Buskmiller C, Ho S, Chen M, Gants S, Crowe E, Lopez S. Patient-centered perinatal palliative care: family birth plans, outcomes, and resource utilization in a diverse cohort. Am J Obstet Gynecol MFM. 2022;4(6):100725. 10.1016/j.ajogmf.2022.100725 35995365

[35] Ng SKF, Keenan N, Swart S, Berry MJ. Palliative care in a tertiary neonatal intensive care unit: a 10-year review. BMJ Support Palliat Care. 2022;12(e5):e641–e645. 10.1136/bmjspcare-2018-001538 30470701

[36] Coney T, Russell R, Palatnik A. 371: Maternal outcomes of pregnancies complicated by fetal life-limiting or lethal conditions. Am J Obstet Gynecol. 2020;222(1):S246–S247. 10.1016/j.ajog.2019.11.387

[37] Han Z, Chen X, Wang Q, et al Clinical characteristics and risk factors of mirror syndrome: a retrospective case-control study. BMC Pregnancy Childbirth. 2021;21(1):581. 10.1186/s12884-021-04143-3 34583666 PMC8480018

[38] Corbetta-Rastelli CM, Friedman AM, Sobhani NC, Arditi B, Goffman D, Wen T. Postpartum hemorrhage trends and outcomes in the United States, 2000–2019. Obstet Gynecol. 2023;141(1):152–161. 10.1097/AOG.0000000000004972 36701615

[39] Kerns JL, Brown K, Nippita S, Steinauer J. Society of family planning clinical recommendation: management of hemorrhage at the time of abortion. Contraception. 2024;129:110292. 10.1016/j.contraception.2023.110292 37739302

[40] Gibson C . Florida abortion ban leaves woman carrying unviable pregnancy to term. The Washington Post. Updated February 18, 2023. Accessed February 12, 2025. https://www.washingtonpost.com/health/2023/02/18/florida-abortion-ban-unviable-pregnancy-potter-syndrome/

[41] Guttmacher Institute . Induced Abortion in the United States. Guttmacher Institute; 2023.

[42] National Coalition for Hospice and Palliative Care . Coalition statement: recent Supreme Court decision, Dobbs vs. Jackson. 2025. Accessed February 12, 2025. https://www.nationalcoalitionhpc.org/coalition-statement-recent-supreme-court-decision-dobbs-vs-jackson

[43] Denney-Koelsch E . 4 ways palliative care can move forward in the post-Dobbs era. Center to Advance Palliative Care. 2024. Accessed February 12, 2025. https://www.capc.org/blog/4-ways-palliative-care-can-move-forward-in-the-post-dobbs-era/

[44] US Congress . S.6—Born-Alive Abortion Survivors Protection Act. 2025. Accessed February 12, 2025. https://www.congress.gov/bill/119th-congress/senate-bill/6/text

