# Supplementary material for: Dobbs-driven expansion of perinatal palliative care: a scoping review of the evidence and its limits
Source: Health Aff Sch. 2025 Apr 15;3(5):qxaf081. doi: 10.1093/haschl/qxaf081 (PMC12043006; doi:10.1093/haschl/qxaf081)
Supplement: qxaf081_Supplementary_Data [file qxaf081_supplementary_data.zip › Appendix A- Search Histories.docx]

APPENDIX

OVID Medline(R) ALL

| 1 | exp Congenital Abnormalities/ or exp Anencephaly/ or exp Trisomy 13 Syndrome/ or exp Trisomy 18 Syndrome/ or exp Abnormalities, Multiple/ or exp Heart Defects, Congenital/ or exp Hernias, Diaphragmatic, Congenital/ or exp Neural Tube Defects/ or exp Encephalocele/ or exp Triploidy/ or exp Polycystic Kidney Diseases/ or exp Thanatophoric Dysplasia/ |
| --- | --- |
| 2 | (life-limiting and (condition* or diagnos*)).mp. |
| 3 | ((life-limiting or life-threatening) adj (condition* or diagnos*)).mp. |
| 4 | ((lethal or structural or congenital) adj (anomal* or abnormal*)).mp. |
| 5 | (Anencephaly or Trisomy or Patau or Edwards syndrome or Bilateral renal agenesis or potters syndrome or severe heart defects or congenital diaphragmatic hernia or Acrania or Encephalocele or Triploidy or Polycystic kidney disease or Thanatophoric dwarfism).mp. |
| 6 | or/1-5 |
| 7 | exp Infant, Newborn/ or Fetus/ |
| 8 | (infant* or newborn* or neonat* or newly born or newly-born).mp. or new-born*.ti,ab. |
| 9 | (Foetus or Fetus or Foetal).mp. or Fetal.ti,ab. |
| 10 | or/7-9 |
| 11 | exp Hospice Care/ or exp Palliative Care/ or exp Fetal Death/ |
| 12 | (infant death or Hospice Care or Palliative Care or Fetal Death*).mp. |
| 13 | (perinatal adj (hospice or palliative or loss)).mp. |
| 14 | 11 or 12 or 13 |
| 15 | 6 and 10 and 14 |

OVID EMBASE

| 1 | exp Congenital Abnormalities/ or exp Anencephaly/ or exp Trisomy 13 Syndrome/ or exp Trisomy 18 Syndrome/ or exp Abnormalities, Multiple/ or exp Heart Defects, Congenital/ or exp Hernias, Diaphragmatic, Congenital/ or exp Neural Tube Defects/ or exp Encephalocele/ or exp Triploidy/ or exp Polycystic Kidney Diseases/ or exp Thanatophoric Dysplasia/ |
| --- | --- |
| 2 | (life-limiting and (condition* or diagnos*)).mp. |
| 3 | ((life-limiting or life-threatening) adj (condition* or diagnos*)).mp. |
| 4 | ((lethal or structural or congenital) adj (anomal* or abnormal*)).mp. |
| 5 | (Anencephaly or Trisomy or Patau or Edwards syndrome or Bilateral renal agenesis or potters syndrome or severe heart defects or congenital diaphragmatic hernia or Acrania or Encephalocele or Triploidy or Polycystic kidney disease or Thanatophoric dwarfism).mp. |
| 6 | or/1-5 |
| 7 | exp Infant, Newborn/ or Fetus/ |
| 8 | (infant* or newborn* or neonat* or newly born or newly-born).mp. or new-born*.ti,ab. |
| 9 | (Foetus or Fetus or Foetal).mp. or Fetal.ti,ab. |
| 10 | or/7-9 |
| 11 | exp Hospice Care/ or exp Palliative Care/ or exp Fetal Death/ |
| 12 | (infant death or Hospice Care or Palliative Care or Fetal Death*).mp. |
| 13 | (perinatal adj (hospice or palliative or loss)).mp. |
| 14 | 11 or 12 or 13 |
| 15 | 6 and 10 and 14 |
| 16 | limit 15 to "remove medline records" |

OVID APA PsycInfo

| 1 | exp Congenital Abnormalities/ or exp Anencephaly/ |
| --- | --- |
| 2 | (life-limiting and (condition* or diagnos*)).mp. |
| 3 | ((life-limiting or life-threatening) adj (condition* or diagnos*)).mp. |
| 4 | ((lethal or structural or congenital) adj (anomal* or abnormal*)).mp. |
| 5 | (Anencephaly or Trisomy or Patau or Edwards syndrome or Bilateral renal agenesis or potters syndrome or severe heart defects or congenital diaphragmatic hernia or Acrania or Encephalocele or Triploidy or Polycystic kidney disease or Thanatophoric dwarfism).mp. |
| 6 | or/1-5 |
| 7 | exp Infant, Newborn/ or Fetus/ |
| 8 | (infant* or newborn* or neonat* or newly born or newly-born).mp. or new-born*.ti,ab. |
| 9 | (Foetus or Fetus or Foetal).mp. or Fetal.ti,ab. |
| 10 | or/7-9 |
| 11 | exp Hospice/ or exp Palliative Care/ |
| 12 | (infant death or Hospice Care or Palliative Care or Fetal Death*).mp. |
| 13 | (perinatal adj (hospice or palliative or loss)).mp. |
| 14 | 11 or 12 or 13 |
| 15 | 6 and 10 and 14 |

Web of Science

| 4 | 1 AND 2 AND 3 |
| --- | --- |
| 3 | **TS=((HOSPICE OR PALLIATIVE OR "FETAL DEATH" OR "FOETAL DEATH" OR "INFANT DEATH"))** |
| 2 | **TS=((NEWBORN OR INFANT OR NEONAT* OR NEWLY-BORN OR NEWLY BORN OR FOETUS OR FETUS OR FETAL OR FOETAL))** |
| 1 | **(((TS=(("congenital abnormalities" OR ANENCEPHALY OR "TRISOMY 13" OR "TRISOMY 18" OR "MULTIPLE ABNORMALITIES" OR CONGENITAL HEAT DEFECTS OR CONGENITAL DIAPHRAGMATIC HERNIA OR NEURAL TUBE DEFECT* OR ENCEPHALOCELE OR TRIPLOIDY OR POLYCYSTIC KIDNEY DISEASE OR THANATOPHORIC DYSPLASIA))) OR TS=((LIFE-LIMITING CONDITION* OR LIFE-LIMITING DIAGNOS*))) OR ALL=(LIFE-THREATENING CONDITION* OR LIFE-THREATENING DIAGNOS*)) OR TS=((LETHAL OR STRUCTURAL OR CONGENITAL) NEAR/3 (ANOMAL* OR ABNORMAL*))** |

CINAHL

| S13 | S4 AND S8 AND S12 |
| --- | --- |
| S12 | S9 OR S10 OR S11 |
| S11 | perinatal N2 (hospice OR palliative OR loss) |
| S10 | infant death OR Hospice Care OR Palliative Care OR Fetal Death* |
| S9 | (MH "Hospice Care") OR (MH "Palliative Care") OR (MH "Perinatal Death") OR (MH "Infant Death+") |
| S8 | S5 OR S6 OR S7 |
| S7 | Foetus OR Fetus OR Foetal OR Fetal |
| S6 | infant* OR newborn* OR neonat* OR newly born OR newly-born OR new-born* |
| S5 | (MH "Infant, Newborn+") OR (MH "Fetus+") |
| S4 | S1 OR S2 OR S3 |
| S3 | Anencephaly OR Trisomy OR Patau OR Edwards syndrome OR Bilateral renal agenesis OR potters syndrome OR severe heart defects OR congenital diaphragmatic hernia OR Acrania OR Encephalocele OR Triploidy OR Polycystic kidney disease OR Thanatophoric dwarfism |
| S2 | life-limiting N2 (condition* OR diagnos*) |
| S1 | MH ("Congenital, Hereditary, and Neonatal Diseases and Abnormalities+") OR (MH "Anencephaly") OR (MH "Trisomy 13") OR (MH "Trisomy 18") OR (MH "Abnormalities, Multiple+")  OR (MH "Heart Defects, Congenital+") OR (MH "Hernia, Diaphragmatic, Congenital")  OR (MH "Neural Tube Defects+") OR (MH "Encephalomyelitis, Acute Disseminated")  OR (MH "Polycystic Kidney, Autosomal Dominant") OR (MH "Polycystic Kidney, Autosomal Recessive") |
